# Prevalence and antimicrobial resistance of *Salmonella enterica* serovars Enteritidis and Typhimurium isolated from retail chicken meat in Wasit markets, Iraq

**DOI:** 10.14202/vetworld.2023.455-463

**Published:** 2023-03-17

**Authors:** Manal H. G. Kanaan

**Affiliations:** Department of Agriculture, Technical Institute of Suwaria, Middle Technical University, Baghdad, Iraq

**Keywords:** antimicrobial resistance, chicken, food poisoning, serovars

## Abstract

**Background and Aim:**

Food poisoning caused by *Salmonella enterica* serovars is the most common type of foodborne illness. Tainted chicken meat is a major vector for spreading these serovars throughout the food supply chain. *Salmonella* isolates that developed resistance to commonly used antimicrobials pose a noteworthy risk to public health, yet there has been a lack of data on this issue in Iraq. Therefore, it is crucial to address these serious public health challenges with an adequate database on the occurrence and antibiotic resistance of these serovars. This study aimed to determine the frequency of occurrence of *Salmonella enterica* serovars Enteritidis and Typhimurium (*S*. Enteritidis and *S*. Typhimurium), antimicrobial resistance (AMR), and prevalence of multidrug resistance among *S*. Enteritidis and *S*. Typhimurium isolated from poultry meat collected in Wasit Province in Iraq.

**Materials and Methods:**

A total of 150 raw and frozen poultry meat samples were gathered from retail markets in various locales across the Wasit Governorate in Iraq. *Salmonella* spp. were successfully cultured and identified using the technique recommended by ISO 6579:2002, with minor modifications. The multiplex polymerase chain reaction approach was used to confirm *Salmonella* spp. (*S*. Enteritidis and *S*. Typhimurium). A disk diffusion test was performed to determine the susceptibility to particular antimicrobial agents, and 12 different antimicrobial agents were evaluated.

**Results:**

Only 19 of the 150 (12.7%) samples tested positive for *Salmonella* (16% and 11% were isolated from raw and frozen chicken meat, respectively). *S*. Enteritidis accounted for 63.2%, whereas *S*. Typhimurium accounted for 36.8%. Nalidixic acid resistance was the most common (73.7%), followed by sulfamethoxazole-trimethoprim (63.2%) and tetracycline (63.2%), but gentamicin and ciprofloxacin (up to 15.8%) only had modest resistance. Antibiogram of *S*. Enteritidis and *S*. Typhimurium yield 13 antibiotypes. Among the 19 *Salmonella* isolates, 12 of 19 (63.2%) established resistance to no less than three categories of antimicrobials.

**Conclusion:**

This study highlighted the necessity of limiting the utilization of antibiotics in animal production by providing vital information regarding the frequency and AMR of *Salmonella* at markets in Wasit Province. Therefore, risk assessment models could use these data to lessen the amount of *Salmonella* passed on to humans in Iraq from chicken meat.

## Introduction

*Salmonella* is a common foodborne bacterium responsible for an estimated 1.3 billion instances of enteric infection and 500,000 deaths due to diarrhea each year worldwide [1]. Nontyphoidal *Salmonella* (NTS) serovars are pathogens of worldwide health relevance, as they are tied to numerous human salmonellosis incidences induced by the consumption of *Salmonella*-contaminated foodstuff of animal sources, such as poultry and derived products, pork, and fish [2, 3]. In particular, *Salmonella* spp. have spread through poultry products, resulting in human and animal illnesses and financial losses. It is impossible to accurately gauge the scope of the problem due to the underreporting of foodborne illnesses worldwide, but the situation is most dire in underdeveloped countries [4]. Because of this, it has been proven difficult to track the origins of epidemics and the agents responsible for their spread. In countries on the path to economic development, wet markets are becoming increasingly significant sources of chicken for local community consumption. At these establishments, the occurrence rates for *Salmonella* spp. in fresh chicken meat contrast greatly from country to country and establishment to establishment, and they can even approach 100% in some cases [5]. Even with substantial progress in technology and hygienic criteria at every stage of the chicken industry, salmonellosis and *Salmonella* outbreaks persist in posing a massive risk to human and animal health [6, 7]. The proliferation of human enteropathogens, such as *Salmonella* strains, is a major cause for concern in Iraq [8, 9]. A survey conducted in Iraq found that *Salmonella* is the second greatest common source of diarrhea in children, after adenovirus, in stool samples taken from those children who had the condition [9].

Most NTS gastroenteritis cases are self-limiting, and symptoms typically disappear within 1 week in healthy patients [10]. However, antimicrobial treatment is necessary after an infection has become invasive in a human and spread to other body parts. This is the case for various systemic infections, including bacteremia, vasculitis, cerebral, lung, and urinary tract infections, and osteomyelitis. Invasive NTS illnesses can be fatal and disproportionately afflict the elderly, infants, and people with immunocompromised systems [11]. Clinicians routinely prescribe third-generation cephalosporins, quinolones, and macrolides to cure human *Salmonella* infections in humans [12]. Misusing antibiotics is a major contributor to the problem of widespread antibiotic resistance. Although antibiotic treatment for systemic infections is often successful, antibiotic resistance is a major health concern [10]. Antibiotic resistance was found in 17%–20% of strains and the prevalence of multidrug resistance (MDR) was increasing, according to a previous report by Scallan *et al*. [13]. Resistance to therapeutically significant antimicrobials, such as fluoroquinolones and third-generation cephalosporins, and the increasing prevalence of MDR *Salmonella* is emerging problems worldwide [3]. The previous study has shown that MDR *Salmonella enterica* can be recognized in various food products, notably meat, and dairy products [14], highlighting that this *Salmonella* strain is being passed from animals to humans via the food supply.

Therefore, addressing the demography of these determinants is crucial to provide effective medicine and illness prevention. This study aimed to shed light on these issues to discover the role of these products in the epidemiology of this foodborne pathogen and guarantee the items’ safety for sale in Wasit Province in Iraq, where only a few comprehensive studies have been conducted.

## Materials and Methods

### Ethical approval

The study was approved by the Technical Institute of Suwaria, Middle Technical University, Baghdad, Iraq. No human or live animal samples were examined in this study. All methods were carried out in accordance with relevant guidelines and regulations.

### Study period and location

The study was conducted from December 2021 to March 2022. All of the samples were procured from various locations within the Wasit markets, and the samples were inspected at the Technical Institute of Suwaria in Wasit province.

### Sample collection

A total of 150 raw and frozen poultry meat samples, including raw chicken meat samples (n = 50) and frozen chicken meat samples (n = 100), were purchased randomly from various native marketplaces and retailers in Wasit Governorate in Iraq for analysis. The samples were packaged in sealed plastic bags, chilled on the way to the laboratory, and then processed <3 h after receiving them.

### Bacterial isolation and identification

Sample analysis and identification of the organism were performed based on the technique recommended by ISO 6579 [15] and as earlier designated by Kebede *et al*. [16], with slight modifications. Briefly, 450 mL tryptone soy broth (TSB) (Oxoid, CM0129, UK) containing 0.6% yeast extract (TSB-YE) and 50 g of each sample were placed inside a stomacher and mashed for 2 min. The mixture was incubated at 37°C for 18 h. After that, 9 mL tetrathionate broth (Oxoid, CM0029) and 1 mL pre-enrichment culture were mixed well and incubated at 37°C for 18 h. A loop-full of culture was applied to the surfaces of xylose lysine deoxycholate (XLD, Oxoid, CM0469) and brilliant green agar (BGA, Oxoid, CM0263) and incubated at 37°C for 1–2 days. On XLD and BGA plates, the formation of pinkish colonies with bright red borders and red colonies with black centers was analyzed, respectively [15]. The purified suspicious colonies were grown at 37°C for 18 h on nutrient agar (Oxoid, CM0003B). Purified colonies underwent additional confirmation using Gram staining, triple sugar iron (TSI), citrate, urease, and indole tests, and conventional biochemical analyses [16].

Additional biochemical tests were conducted using Oxoid™ biochemical identification system *Salmonella* (Oxoid, ID0570) to differentiate *Salmonella* spp. from other organisms that showed a similar colonial appearance on a common selective *Salmonella* medium. *Salmonella* isolates were discriminated by screening for the enzymes pyroglutamyl aminopeptidase (PYRase) and nitrophenylalanine deaminase (NPA). The *Salmonella* isolates were frozen at −80°C in a double-concentration TSB-YE broth containing 20% (v/v) pure medicinal glycerin so that further study could be done [17].

### Confirmation of *Salmonella* isolates through the multiplex polymerase chain reaction (mPCR) technique

To extract deoxyribonucleic acid (DNA), stock cultures of *Salmonella* isolates were melted overnight at 4°C, invigorated on tryptone soy agar (TSA, Oxoid, CM0131), and incubated overnight at 35°C ± 2°C [18]. Finally, the isolates were cultured on TSB for an additional overnight. Afterward, the Wizard^®^ Genomic DNA Extraction and Purification Kit (Promega, United States) were used to extract and purify the bacterial genome. An mPCR technique based on primers developed by Saeki *et al*. [19] was used to detect *S. enterica* serovars simultaneously. The primers amplified *Salmonella* spp., *Salmonella enterica* serovar Enteritidis (*Salmonella* Enteritidis), and *Salmonella enterica* serovar Typhimurium (*Salmonella* Typhimurium) fragments of 199, 299, and 759 bp, respectively (Table-1) [19]. The ultimate volume of mPCR was 20 μL; each reaction included 4 μL DNA template, 2 μL PCR buffer (20 mM Tris-HCl, 50 mM KCl, pH 8.4), 4 mM MgCl_2_, 0.6 mM dNTP, 0.3 mM each of Styinva-JHO-2-left and Styinva-JHO-2-right, 0.4 mM ENTF and ENTR, and 0.3 mM each of STM4492F and STM4492R, and 1 μL Taq DNA polymerase (Thermo Scientific, USA) [19]. A preliminary denaturation step at 95°C for 10 min was followed by 35 cycles of denaturation at 94°C for 60 s, 90 s at 60° C, and 90 s at 72°C and a final extension at 72°C for 10 min in a Perkin-Elmer thermocycler system. The products were subjected to 1.5% (w/v) agarose gel electrophoresis at 100 V for 40 min with 0.02 μL/mL SYBR® Safe DNA gel dye, a UV transilluminator, was used to see the bands (Alpha Imager HP, Alpha Innotech, CA, USA). Positive controls were DNA from reference strains, and negative controls were sterile distilled water.

**Table-1:** Primers sequences used in the multiplex polymerase chain reaction assay [19].

Organism	Primers	Sequence of primers (5′–3′)	Target gens	Size in bp
*Salmonella* spp.	Styinva-JHO-2R	AAA CGT TGA AAA ACT GAG GA	*inv*A	199 bp
	Styinva-JHO-2F	TCG TCA TTC CAT TAC CTA CC		
*Salmonella* Enteritidis	ENTR	AAA TGT GTT TTA TCT GAT GCA AGA GG	*sdf*	299 bp
	ENTF	GTT CGT TCT TCT GGT ACT TAC GAT GAC		
*Salmonella* Typhimurium	STM4492R	ACA GCT TGG CCT ACG CGA G	STM4492	759 bp
	STM4492F	AGC AAC CGT TCG GCC TGA C		

### Determination of antimicrobial susceptibility of *Salmonella*

The Kirby–Bauer disk diffusion method has been applied on Mueller-Hinton agar (MHA, Oxoid, CM0337) to investigate the susceptibility of *Salmonella* isolates to various antimicrobial agents in accordance with the Clinical and Laboratory Standards Institute (CLSI) [20] with the following agents: amikacin 10 μg, gentamicin (GEN) 10 μg, cefoxitin 30 μg, ceftriaxone, cefotaxime/clavulanic acid 40 μg, sulfamethoxazole-trimethoprim (COT) 30 μg, aztreonam (ATM) 30 μg, ampicillin (AMP) 10 μg, chloramphenicol (CHL) 30 μg, ciprofloxacin (CIP) 30 μg, nalidixic acid 30 μg, and tetracycline (TET) 30 μg. In brief, bacterial isolates were stored at −80°C in pure medical glycerin, thawed at 4°C, and resuscitated at 35°C ± 2°C on TSA (Oxoid, CM0131) for 18 h. Tryptone soy broth was inoculated with pure colonies and incubated for 4 h at 35°C. Each inoculum’s turbidity was adjusted to meet the 0.5 McFarland standard. Swabs were used to inoculate MHA (Oxoid, CM0337) with *Salmonella* isolates; after 30 min at room temperature to allow drying at 37°C, antibiotic discs were dispensed over the agar. Results were interpreted according to the CLSI zone diameter breakpoints to categorize all isolates as susceptible, intermediate, or resistant [20].

### Statistical analysis

MedCalc version 18 (https://www.medcalc.org/) was used for the statistical evaluation of the data. Two samples were analyzed using the χ^2^ test at the 5% significance level to assess if the difference in proportions is statistically significant.

## Results

Only 19 of the 150 (12.7%) samples could be isolated as credible *Salmonella* spp. based on morphological traits, microscopy, and conventional biochemical inspection (Table-2). Each isolate displayed a unique pattern of colony color and shape when tested using various methods: Gram-negative, black colonies or colonies with black centers and red medium on TSI agar; blue color for the citrate test; purple-red color for the urease test; violet-colored colonies for the indole test; and red colonies with black centers and pink colonies with a red zone when inspected on XLD and BGA plates, respectively [16].

**Table-2:** Prevalence of *Salmonella* spp. in poultry meat retailed in Wasit markets.

Sample’s type	No. of samples analyzed	n/N (%) *Salmonella* spp.	n/N (%) *Salmonella* Enteritidis	n/N (%) *Salmonella* Typhimurium
Raw chicken meat	50	8/50 (16)	5/8 (62.5)	3/8 (37.5)
Frozen chicken meat	100	11/100 (11)	7/11 (63.6)	4/11 (36.4)
Total	150	19/150 (12.7)	12/19 (63.2)	7/19 (36.8)
p-value		p=0.3870	p=0.1083	

Oxoid™ biochemical identification system *Salmonella* revealed that these isolates were negative for Gram lysis state and PYRase and NPA activity. *Salmonella* spp. were isolated at 16% in raw chicken and 11% in frozen chicken (Table-2). Nineteen of the presumed isolates were proven to be *Salmonella* spp. through mPCR testing, with the majority (63.2%) being *S*. Enteritidis and the minority (36.6%) being *S*. Typhimurium. Statistically, the type of sample (i.e., raw and frozen) did not significantly (p > 0.05) affect the prevalence of the pathogen (χ^2^ = 0.748, p = 0.3870) or its serovars (χ^2^ = 2.579, p = 0.1083).

### Antibiotic resistance

**Figure-1:**
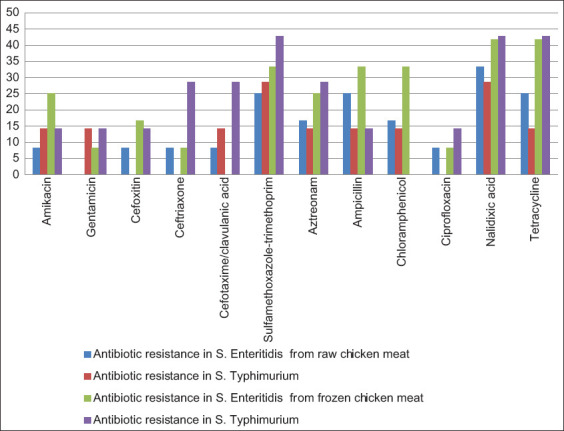
Prevalence of antibiotic resistance in *Salmonella enterica* recovered from retail chicken meat.

Most tested strains demonstrated resistance to NAL, COT, and TET with rates of 73.7%, 63.2%, and 63.2%, respectively (Table-3). In contrast, a low resistance rate was detected against GEN and CIP (15.8%). Furthermore, concerning *S. enterica* serovars and irrespective of the sample category, *S*. Enteritidis had the highest resistance rate to NAL, TET, AMP, and COT (58.3%–75%), whereas *S*. Typhimurium exhibited high resistance to NAL, TET, and COT (57.1%–71.4%; Table-3; Figure-1). However, regarding the sample category, frozen chicken isolates exhibited resistance rates similar to raw chicken isolates against the selected antimicrobials (Figure-1). Statistically, there was no significant influence (p > 0.05) on the sample type on the resistance to antimicrobials (Table-3).

**Table-3:** Prevalence of antibiotic resistance in *Salmonella* *enterica* Enteritidis and Typhimurium recovered from retail poultry meat in Wasit marketplaces.

Antibiotics	No. (%) of resistant isolates					

	Sample's type					
	Raw chicken meat		Frozen chicken meat		Total (n=19)	p-value
	
	*S.* Enteritidis (n=5)	*S.* Typhimurium (n=3)	*S.* Enteritidis (n=7)	*S.* Typhimurium (n=4)		
Amikacin	1 (20)	1 (33.3)	3 (53.3)	1 (25)	6 (31.6)	0.3768
Gentamicin	0 (0)	1 (33.3)	1 (14.3)	1 (25)	3 (15.8)	0.5577
Cefoxitin	1 (20)	0 (0)	2 (28.6)	1 (25)	4 (21.1)	0.2986
Ceftriaxone	1 (20)	0 (0)	1 (14.3)	2 (50)	4 (21.1)	0.2986
Cefotaxime/ clavulanic acid	1 (20)	1 (33.3)	0 (0)	2 (50)	4 (21.1)	1.0000
Sulfamethoxazole - trimethoprim	3 (60)	2 (66.7)	4 (57.1)	3 (75)	12 (63.2)	0.4920
Aztreonam	2 (40)	1 (33.3)	3 (42.9)	2 (50)	8 (42.1)	0.4334
Ampicillin	3 (60)	1 (33.3)	4 (57.1)	1 (25)	9 (47.4)	0.7100
Chloramphenicol	2 (40)	1 (33.3)	4 (57.1)	0 (0)	7 (36.8)	0.6777
Ciprofloxacin	1 (20)	0 (0)	1 (14.3)	1 (25)	3 (15.8)	0.5577
Nalidixic acid	4 (80)	2 (66.7)	5 (71.4)	3 (75)	14 (73.7)	0.5080
Tetracycline	3 (60)	1 (33.3)	5 (71.4)	3 (75)	12 (63.2)	0.1695

*S.* Enteritidis*=Salmonella enterica* serovar Enteritidis, *S.* Typhimurium=*Salmonella enterica* serovar Typhimurium

The Antibiogram and MDR index of *S*. Enteritidis and *S*. Typhimurium are shown in Table-4. These isolates produced 13 antibiotypes in 10 antibiogroups according to the number of drugs that every isolate displayed resistance to them. Notably, most tested isolates (12/19 or 63.2% of the total) developed resistance to at least three drugs, whereas 47.4% showed resistance to no less than six antibiotics. Multidrug resistance was also found in 62.5% of raw chicken meat isolates and 63.6% of frozen chicken meat isolates. Statistically and based on the sample category, there was no significant influence (p > 0.05) on this phenomenon in these isolates (χ^2^ = 0.002, p = 0.9619).

**Table-4:** Antibiogram and MAR index of *Salmonella enterica* Enteritidis and Typhimurium recovered from retail poultry meat in Wasit marketplaces.

Antibiotypes	No. of antimicrobials	No. (%) of experienced isolates					Total (n = 19)

		Sample’s type					

		Raw chicken meat		Frozen chicken meat		Antibiogroups	
	
		*S.* Enteritidis (n = 5)	*S.* Typhimurium (n = 3)	*S.* Enteritidis (n = 7)	*S.* Typhimurium (n = 4)		
(AMI, FOX, AXO, CTC, COT, ATM, AMP, CHL, CIP, ND, TET)	11	1 (20)	0 (0)	0 (0)	0 (0)	1 A	3 (15.8)
(AMI, GEN, FOX, AXO, COT, ATM, AMP, CHL, CIP, ND, TET)	11	0 (0)	0 (0)	1 (14.3)	0 (0)	1 B	
(AMI, GEN, FOX, AXO, CTC, COT, ATM, AMP, CIP, ND, TET)	11	0 (0)	0 (0)	0 (0)	1 (25)	1 C	
(AMI, GEN, AXO, CTC, COT, ATM, AMP, CHL, ND, TET)	10	0 (0)	1 (33.3)	0 (0)	0 (0)	2 A	1 (5.3)
(AMI, FOX, COT, ATM, AMP, CHL, ND, TET)	8	0 (0)	0 (0)	1 (14.3)	0 (0)	3 A	1 (5.3)
(AMI, COT, ATM, AMP, CHL, ND, TET)	7	0 (0)	0 (0)	1 (14.3)	0 (0)	4 A	1 (5.3)
(COT, ATM, AMP, CHL, ND, TET)	6	2 (40)	0 (0)	0 (0)	0 (0)	5 A	3 (15.8)
(AXO, CTC, COT, ATM, ND, TET)	6	0 (0)	0 (0)	0 (0)	1 (25)	5 B	
(COT, AMP, CHL, ND, TET)	5	0 (0)	0 (0)	1 (14.3)	0 (0)	6 A	1 (5.3)
(COT, AMP, ND, TET)	4	1 (20)	0 (0)	0 (0)	0 (0)	7 A	1 (5.3)
(COT, ND, TET)	3	0 (0)	0 (0)	0 (0)	1 (25)	8 A	1 (5.3)
(COT, ND)	2	0 (0)	1 (33.3)	0 (0)	0 (0)	9 A	2 (10.5)
(ND, TET)	2	0 (0)	0 (0)	1 (14.3)	0 (0)	9 B	
ND	1	1 (20)	0 (0)	0 (0)	0 (0)	10 A	1 (5.3)
Sensitive	--	1 (20)	0 (0)	1 (14.3)	2 (50)	--	4 (21.1)
Total ARP (13)		7/8 (87.5)		8/11 (72.7)		10	15 (78.9)

AMI=Amikacin, GEN=Gentamicin, FOX=Cefoxitin, AXO=Ceftriaxone, CTC=Cefotaxime/clavulanic acid, COT=Sulfamethoxazole-trimethoprim, ATM=Aztreonam, AMP=Ampicillin, CHL=Chloramphenicol, CIP=Ciprofloxacin, NA=Nalidixic acid, TET=Tetracycline, *S.* Enteritidis*=Salmonella enterica* serovar Enteritidis, *S.* Typhimurium=*Salmonella enterica* serovar Typhimurium, ARP=Antimicrobial resistance profile

## Discussion

*Salmonella* has long been linked to poultry and related products [21]. In recent years, there has been widespread reporting of the growing prevalence of *Salmonella* and antimicrobial resistance (AMR) in food [22]. This study found that 12.7% of retail chicken meat tested positive for *Salmonella* spp., with isolation rates of 16% and 11% in raw and frozen chicken meat, respectively (Table-2). These findings were consistent with the previous Trinidad findings, which found that the frequency of *Salmonella* spp. isolation from supermarket samples was 19.0%, 8.1%, 0.0%, and 7.6% for chilled complete chickens, chilled chicken portions, frozen complete chickens, and frozen chicken portions, respectively [23].

Results were lower than those reported in Malaysia (100%) [5], China (52.2%) [24], Nepal (38.0%) [25], Vietnam (48.9%) [26], and Bengaluru, India (65.0%) [27]. During the slaughtering process, bacterial contamination is likely from various sources, including dirty equipment, unclean water, and the microbiome of the animals themselves [28]. Water baths during procedures have a washing effect that reduces bacterial burdens but can also enhance cross-contamination across carcasses. Therefore, this should be taken into account [28]. The use of chilled water in retail outlets significantly influences the prevalence of *Salmonella* spp., with a remarkably greater frequency of *Salmonella* spp. (66.7%) in carcasses obtained from markets that consumed chilled water than outlets that did not use chilled water (25.7%), as reported by Khan *et al*. [23]. Stagnant water in the chilling water bath can lead to bacterial growth, such as *Salmonella* spp., which can contaminate and cross-contaminate the carcasses if the water is not replaced regularly [23]. In addition, the persistence of *Salmonella* spp. in various sites in processing plants may be attributed to biofilm formation by these bacteria, leading to their widespread dissemination in this environment [29–31]. In the markets, poultry is slaughtered and prepared in small outlets, so reducing the successive steps that carcasses pass through may subsequently reduce the chances of cross-contamination among carcasses. In addition, the low prevalence rates of these bacteria may be related to the time of the study, as it took place within certain months and extended from winter to the beginning of spring, which did not cover the hot summer months. It also explored the prevalence rate of these bacteria within one governorate, reflecting low prevalence rates related to the isolation conditions.

Lower isolation frequencies of up to 1.8% were previously attained by Kebede *et al*. [16]. *Salmonella* contamination of supermarket chicken varies substantially between countries. Possible contributors to this discrepancy include changes in sample size, sample type (whole birds vs. parts, raw meat, refrigerated vs. frozen), sample strategy, chicken breed, and *Salmonella* detection technique [32].

Results revealed a significant manifestation of *Salmonella* in raw chicken meat compared to frozen chicken meat (Table-2), in accordance with the previous results [23, 24, 33]. The limited bacterial growth in nonselected and selective media can be due to the bacteriostatic effect of low temperatures, which is thought to work by injuring the bacterial cell wall [34]. Furthermore, results suggested a higher incidence of *S*. Enteritidis than *S*. Typhimurium in 63.2% of the samples that tested positive for the pathogen, consistent with the previous studies [2, 34–36].

Antimicrobial resistance in *Salmonella* continues to be a critical public health alarm worldwide. The observation database revealed a remarkable increase in the overall prevalence of resistant *Salmonella* strains to at least one antimicrobial agent [33], and this study encouraged this finding.

Results revealed that most isolates exhibited resistance to NAL, COT, and TET with a prevalence of 63.2% to 73.7%, which was nearly similar to the Myanmar findings of chicken meat-resistant isolates to COT up to 70.3% [37] but higher than the resistance in *Salmonella* isolated from raw poultry to COT in China as 56.6% [33]. High resistance to NAL and TET has previously been recorded in Korea [38] and Pakistan [39]. Results also revealed somewhat high resistance to AMP, ATM, and CHL, ranging from 36.8% to 47.4%, and high resistance to AMP and CHL was previously reported [33, 38, 39]. In contrast, in their study, Gelinski *et al*. [40] reported lower resistance to ATM by up to 10%.

Previous findings from China [33] and Brazil [40] showed up to 21.1% resistance against cephalosporins, but higher than that obtained in Korea [38]. They reported that 11.3% of *Salmonella* isolates were resistant to cephalosporins. Antimicrobial resistance is a multifaceted problem with many potential causes, including but not limited to the prevalent utilization of antibiotics in animal rearing for human consumption [41]. Widespread usage of enrofloxacin and norfloxacin in chicken husbandry has been linked to increased fluoroquinolone-resistant bacteria [41]. Extensive utilization of TET in human and veterinary care, also in poultry and cattle feedstuff, has been linked to an uptick of highly resistant microbes [42].

Antibiotic-resistant bacteria have become more common in veterinary and human medicine due to the overuse of β-lactam antibiotics for treating *Salmonella* infections [40]. Despite the ban on pharmaceuticals in animal feed, extensive usage of antibiotics in chicken farming persists due to generics for use in food and water that are significantly more inexpensive than primary commodities [40]. Biofilm generation is a mechanism for achieving AMR and is associated with an elevated risk to food safety [43]. Environmental microorganisms possibly play a crucial role in transferring AMR genes to pathogenic microorganisms, directly affecting human health by entering the food chain. This is because environmental microbes are great sources of AMR genes, also known as “environmental resistomes” [41].

Results showed that most isolates showed low resistance to GEN and CIP, up to (15.8%), in line with the previous findings [16, 38–40]. In contrast, the results were lower than those of Yang *et al*. [33] in China. They reported that 35% of *Salmonella* isolates from raw poultry meat were resistant to GEN.

Apramycin has been employed expansively in veterinary medicine, most likely correlated to the intensification of resistance to GEN [12]. Resistance to cephalosporins can be achieved by overproducing cephalosporinases, enzymes that degrade cephalosporins [44]. Furthermore, results displayed that *S*. Enteritidis had the maximum resistance rate to NAL, TET, AMP, and COT (58.3%–75%), whereas *S*. Typhimurium exhibited high resistance to NAL, TET, and COT (57.1%–71.4%; Table-3; Figure-1). However, regarding the sample type, the resistance rates to the antimicrobials were comparably similar across isolates from raw and frozen chicken (Figure-1), in line with the previous results that showed a variation in resistance according to the serovars of the organism or sample types [33, 38].

Disparities in antimicrobial classes, antibiotics utilization, bacterial serovars, and country of origin of the resulting isolates have all been linked to variances in resistance and susceptibility proportions [21].

The over-application of antimicrobials has given rise to the interference of the balance of the ecosystem, thus creating the enrichment of MDR bacteria [45, 46]. Antibiotic resistance spreads throughout *S. enterica* spp. largely due to horizontal gene transfer between resistant plasmids and bacterial chromosomes. In addition, the conjugation processes promote the transfer of resistance genes from plasmids to other strains and species through transposon or integron [47]. Antimicrobial resistance methods are facilitated by *Salmonella*’s production of enzymes that can harm the activity of antibiotics, activation of efflux pumps, and production of β-lactamase, which can destroy the structure of antibiotic molecules [43]. There has been an increase in the prevalence of extended-spectrum β-lactamase-producing *Salmonella* serotypes, which are resistant to an extensive variety of antibiotics [40].

The most noteworthy finding of this study was that 63.2% of the isolates developed resistance to not less than three antimicrobials, with 47.4% displaying resistance to at least six medicines. Our results are similar to the study conducted in Ethiopia [16] but greater than that conducted in Korea [38]. In Iraq, there is a lack of information on the utilization of antibiotics in animal production. Therefore, this investigation tried to provide useful data to understand the connection between the utilization of antimicrobial agents in poultry production and the upsurge of the emergence of resistance in foodborne pathogens, such as *Salmonella*. The previous studies in Iraq confirmed this relationship by investigating the MDR phenomenon in many foodborne pathogens [21, 48–51]. Other studies in Iraq also pointed to wrong practices regarding the abuse and misuse of antibiotics, which drove the exacerbation of this phenomenon [52–54]. Accordingly, these results highlighted the importance of preventing antibiotic overuse in reducing the MDR of *Salmonella* spp.

## Conclusion

Results revealed some remarkable observations for *S. enterica* serovar Enteritidis and Typhimurium across the food chain. The occurrence of these serovars in meat poses a risk to human health, as it may lead to serious illnesses. The recent diversity of AMR observed among these isolates is of major importance and has considerably attracted attention to antibiotic misuse in recent years. This has occurred as a direct result of antibiotic abuse. Major resistance forms found were 6 and 11 antimicrobials, in which 31.6% of the tested isolates exhibited these patterns. These findings emphasized the need to conduct additional investigations on the presence, distribution, and MDR of *Salmonella* spp. in human food consumed in various geographical locations, spanning more provinces in Iraq, to offer an additional database on this foodborne bacterium. In addition, to lessen the likelihood of cross-contamination and its negative effects on public health, it is important to educate farmers, processors, retailers, and consumers on the dangers of *Salmonella*.

##  Authors’ Contributions

MHGK: Responsible for all aspects of the current investigation, including the study proposal, sample collection, laboratory work, manuscript writing, data analysis, and edits. She checked the final draft and gave her approval.
